# Multicentre outcomes of total hip arthroplasty using a novel collared cementless femoral stem

**DOI:** 10.1302/2633-1462.73.BJO-2025-0233.R1

**Published:** 2026-03-13

**Authors:** John Mahon, Carl Keogh, Behnazir Mohamed, Victoria Byrne, Fergal Moran, Fiachra Rowan, Gerard A. Sheridan, James P. Cashman

**Affiliations:** 1 National Orthopaedic Hospital Cappagh, Dublin, Ireland; 2 University Hospital Galway, Galway, Ireland; 3 Bon Secours Hospital, Galway, Ireland; 4 University Hospital Waterford, Ballynakill, Waterford, Ireland

**Keywords:** Arthroplasty, Femoral stem, Collar, Total hip arthroplasty, Periprosthetic fracture, Revision, Uncemented, Cementless, Actis, Total hip arthroplasty (THA), cementless femoral stem, femoral components, revision surgery, EQ-5D scores, patient-reported outcome measures (PROMs), periprosthetic fracture, cementless femoral components, functional outcome scores, Oxford Hip Score (OHS)

## Abstract

**Aims:**

Recent years have seen increased interest in tissue-sparing approaches for total hip arthroplasty (THA), which has led to innovations in implant design. Short cementless femoral components have gained traction, and the inclusion of a medial calcar collar to improve stability may offset the risk of fracture. The aim of this current study is to report short-term outcomes and survivorship for a novel design of femoral component across four non-designer centres.

**Methods:**

All patients undergoing primary THA across four centres from July 2020 to January 2025 were eligible for inclusion. Data were collected prospectively in a national arthroplasty register, with planned routine follow-up at six months and two years. Patient-reported outcome measures were assessed using the Oxford Hip Score (OHS) and EuroQol five-dimension questionnaire (EQ-5D) score.

**Results:**

A total of 517 components in 489 patients were included in the dataset: three patients (0.6%) died by final follow-up, and of the remaining 514 components, 512 components (99.6%) remain in situ. For the two patients (0.4%) undergoing revision surgery, indications for revision were periprosthetic fracture (PPF) and large postoperative haematoma. PPF affected four patients (0.8%) in total: two intraoperative events were managed with cables, and one Vancouver C fracture was managed with plate and screw fixation. The mean preoperative OHS was 17 (95% CI 16.3 to 17.7) with a mean postoperative score of 40.7 (95% CI 39.7 to 41.5), and mean preoperative EQ-5D score was 0.36 (95% CI 0.34 to 0.38), with a mean postoperative score of 0.80 (95% CI 0.78 to 0.82).

**Conclusion:**

This novel femoral component demonstrates excellent functional outcomes which are reproducible across multiple surgeons in non-designer centres, with low rates of revision surgery and PPF.

Cite this article: *Bone Jt Open* 2026;7(3):366–372.

## Introduction

Total hip arthroplasty (THA) is a common and successful procedure, to the extent that it has been described as ‘the operation of the century’.^1,2^ Data from the UK and USA show a year-on-year increase in the volume of THAs performed, with the exception of the COVID-19 pandemic in 2020.^3,4^ This is commensurate with service expansion and ageing population demographic details; however, with greater numbers of procedures performed, the burden of morbidity also increases. Additionally, there has been a trend towards increasing usage of cementless components, which historically have been associated with an increased risk of periprosthetic fracture (PPF) relative to cemented components.^3,5-7^

More recent research suggests that cementless femoral component designs featuring a medial calcar collar may offset the risk of PPF, and some series have shown a significantly lower rate of postoperative PPF (POPPF) and reoperation compared with cemented components.^8-11^

In the context of increasing rates of THA and continued evolution of surgical practice, large-scale databases such as arthroplasty registries are useful for identifying trends and optimizing practice.^12^ Multiple factors contribute to heterogeneity of this data, including construct modularity and variations in surgical practice, which makes it more difficult to interpret and draw meaningful conclusions.

Ongoing developments in biomaterials technology and manufacturing processes have contributed to advancements in THA.^13^ There is utility in the concept of a ‘workhorse’ femoral component which takes advantage of these innovations to reduce surgical morbidity and optimize compatibility across surgical factors, such as choice of approach, and patient factors, such as age and sex.

The Actis (DePuy Synthes, USA) cementless femoral component was developed in 2015. It is a fully hydroxyapatite (HA) coated, triple-tapered short component with a medial collar, designed for compatibility with tissue-sparing approaches.^14^ The aim of this study is to report multicentre, non-designer outcomes and survivorship for the Actis component using a posterior approach at short-term follow-up.

## Methods

This was a retrospective review of prospectively collected registry data conducted across four centres (National Orthopaedic Hospital Cappagh, University Hospital Galway, Bon Secours Hospital Galway, and University Hospital Waterford). We retrospectively reviewed our national arthroplasty registry for patients undergoing primary THA between July 2020 and January 2025. All patients that received the Actis component were eligible for inclusion. Exclusion criteria were THA for malignancy or fracture.

The primary outcomes were defined as the number of components in situ, death occurring during the follow-up period with components in situ, and all-cause reoperation or revision surgery. Secondary outcomes were pre- and postoperative patient-reported outcome measures (PROMs), namely the Oxford Hip Score (OHS)^15-17^ as a joint-specific measure of pain and disability with scores ranging from 0 (worst) to 48 (best), and the EuroQol five-dimension questionnaire (EQ-5D)^18^ score as a concise measure of general health and estimation of quality-adjusted life-years, in which a score of 1 indicates ‘full health’ and 0 would correspond to death.

Demographic information collected included patient sex, age, and BMI. Data collection was conducted by review of patient and operative records. Implant details were available from the joint registry.

### Patient demographic details

A total of 517 components in 482 patients were included in the dataset, of whom 221 were female (45.9%). Bilateral THA was performed in 35 patients (7.3%). Of the total cohort, 273 hips (52.8%) were right-sided. The mean patient age was 60.9 years (14 to 94; SD 12.7), with 75 patients (15.6%) aged under 50 years. The mean BMI was 29.8 kg/m^2^ (18 to 49; SD 5.1). Demographic data is summarized in Table I.

**Table I.** Patient demographic details.

Variable	Data
Mean age, yrs (range; SD)	60.9 (14 to 94; 12.7)
Mean BMI, kg/m^2^ (range; SD)	29.8 (18 to 49; 5.1)
**Sex, n (%)**	
Male	261 (54.1)
Female	221 (45.9)
**Side, n (%)**	
Right	273 (52.8)
Left	244 (47.2)

### Statistical analysis

All statistical analysis and graphic generation was performed using RStudio-2025.05.0+496 computer software (Posit, USA), and Excel (Microsoft, USA).

Data were normality tested using the Shapiro-Wilk test with construction of Q-Q plots. The Wilcoxon signed rank test was used for comparison of paired samples of non-parametric data. Box plots were generated to illustrate functional outcome scores. Implant details were represented with a heatmap for comparison of femoral component and cup sizes in combination. Cumulative incidence curves were used to illustrate reoperation event probability. Person-time incidence rates were calculated to clarify time-to-event for complications and reoperation. Statistical significance was set at p < 0.05.

### Ethical approval

This project was reviewed and formally approved by the National Orthopaedic Hospital Research Ethics Committee reference NOHC-2025-ETH-MB-CEO-375. All participants gave informed consent for the use of their data prior to their initial surgery and inclusion in the National Orthopaedic Registry.

## Results

### Implant details

**Fig. 1:**
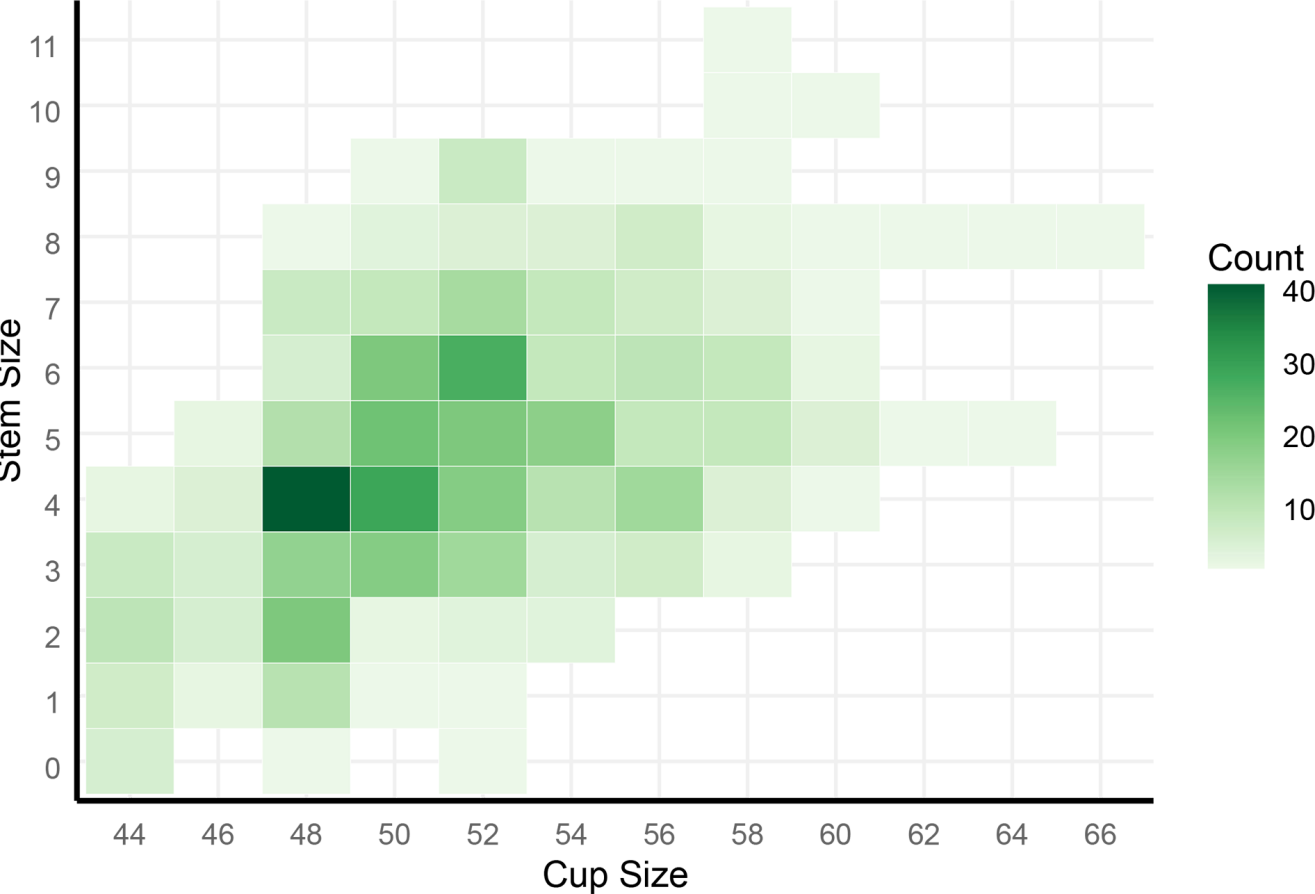
Heatmap of construct size.

All femoral components in our series featured a medial collar: sizes ranged from 0 to 11 with a modal value of 4 (22.4%), and 283 were standard offset (54.7%). All patients received ceramic heads, and head size ranged from 28 mm to 36 mm with modal head size of 32 mm (48.9%). All acetabular components in our series were from the Pinnacle Acetabular System (DePuy Synthes, USA). Cup size ranged from 44 mm to 66 mm with a modal value of 48 mm (21.3%). Screws were used for acetabular fixation in 54 cases (10.4%). A heatmap of cup and femoral component sizes in combination is shown in Figure 1. A neutral liner was used in 393 cases (76%), 71 cases used a lipped liner (13.7%), 39 cases used a Delta Ceramic liner (7.5%), and five cases used a + 4 mm 10° ‘face-changing’ liner (1%).

### Femoral component results

**Fig. 2:**
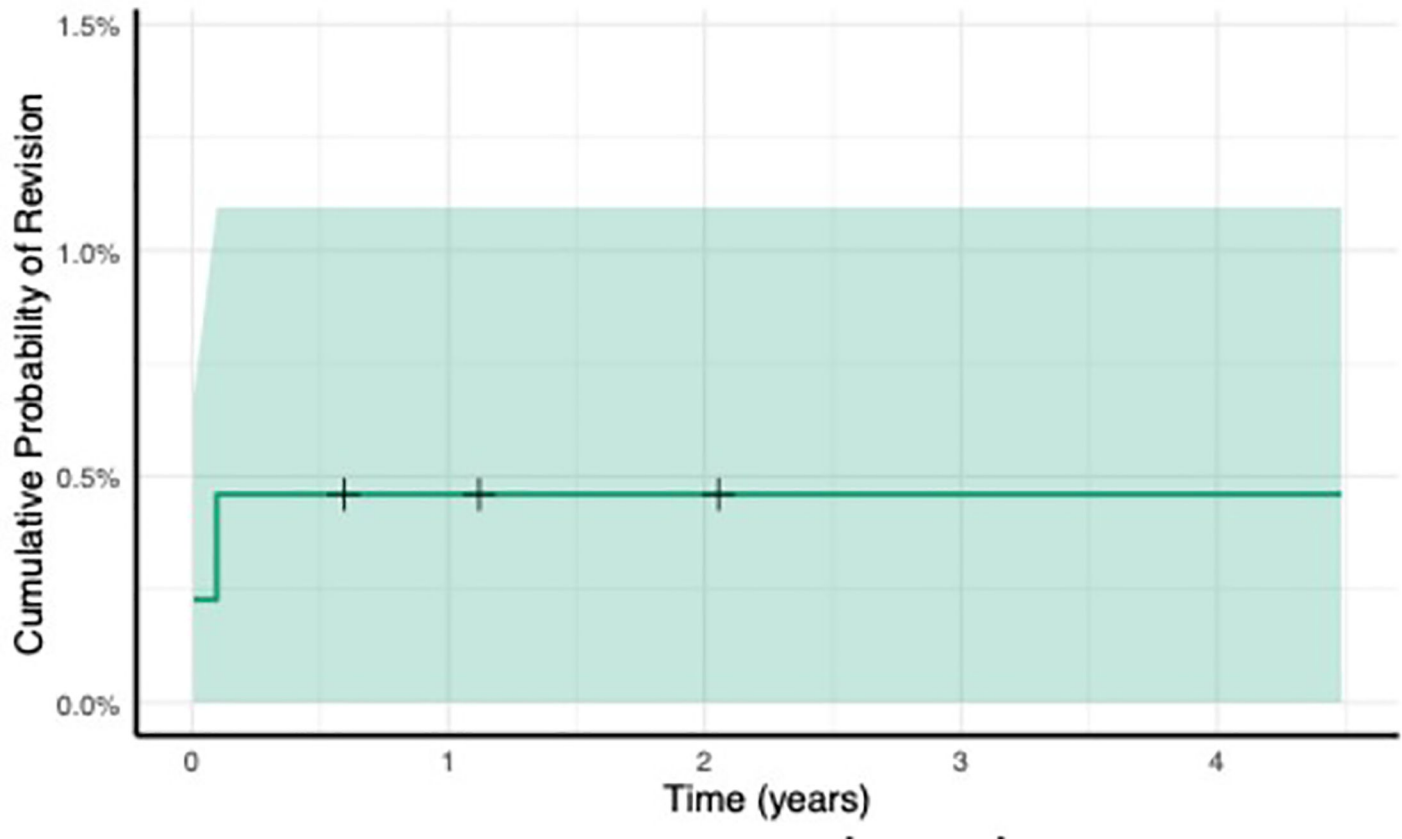
Cumulative incidence of revision (1-Kaplain-Meier curve) (deaths censored).

Three patients (0.6%) died before final follow-up, and of the remaining 514 components, 512 (99.6%) remained in situ at a mean follow-up of 23.1 months (0.2 to 54.6; SD 15.8); a cumulative incidence curve (1-Kaplain-Meier curve) of femoral component revision is shown in Figure 2. Two patients were affected by intraoperative complications, both intraoperative PPF (IOPPF) (0.4%) which were managed with cables and have had a successful postoperative course; both patients reported excellent OHS and EQ-5D scores at six-month follow-up, and both femoral components remain in situ without further complication at 46 months and 19 months.

There were 15 patients affected by postoperative complications (2.9%); five patients underwent reoperation (1%), and two patients had revision of the femoral component (0.4%). One patient underwent revision surgery for an early POPPF on the second postoperative day; it is possible that this represents an unrecognized IOPPF event given its early occurrence. The other revision case returned to theatre one month postoperatively with a large non-resolving haematoma due to anticoagulation. This was undertaken to assess for and treat a potential periprosthetic joint infection (PJI); however, there was no evidence of infection intraoperatively, and intraoperative samples did not culture any pathogen.

There were three patients affected by instability; two of these were isolated dislocation events within two weeks of surgery, managed with closed reduction without further sequelae. The third patient underwent reoperation 11 weeks postoperatively, which found disruption of the short external rotator muscles; they underwent exchange of modular components to a larger 36 mm head with a taper sleeve, and a + 4 mm 10° liner.

One patient was affected by POPPF at 20 months postoperatively, and was managed with open reduction and internal fixation. There were two patients affected by PJI; one underwent debridement with antibiotics and implant retention two months postoperatively and remains asymptomatic and off antibiotics at 14 months follow-up, the other patient was managed with oral antibiotics and passed away due to malignancy. One patient was affected by a limb length discrepancy of < 2 cm which was managed with a shoe raise, four patients experienced wound issues managed by their primary care doctor with oral antibiotics, and two patients required transfer or admission to hospital in the early postoperative period for medical comorbidities. A summary of femoral component outcomes is shown in Table II.

**Fig. 3:**
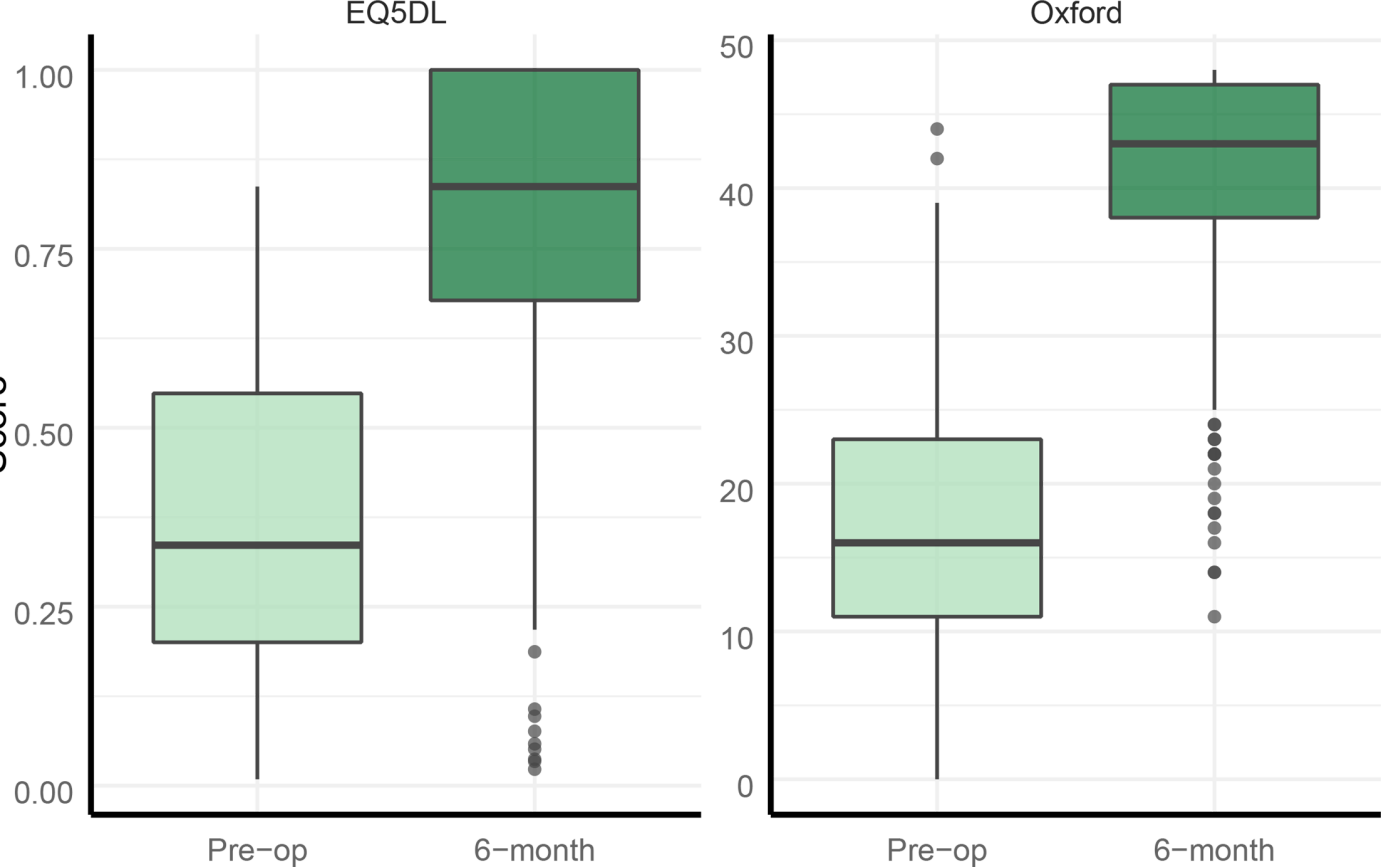
Patient reported outcome measure results. EQ-5D, EuroQol five-dimension questionnaire.

PROMs at six-month follow-up were recorded for 321 femoral components (62.5%). The mean preoperative OHS was 17 (95% CI 16.3 to 17.7) with a mean postoperative score of 40.7 (95% CI 39.7 to 41.5), and mean preoperative EQ-5D score was 0.36 (95% CI 0.34 to 0.38) with a mean postoperative score of 0.80 (95% CI 0.78 to 0.82). Figure 3 represents these results with boxplots. The mean improvements in pre- and postoperative OHS and EQ-5D scores were 23.4 (95% CI 22.2 to 24.5, p < 0.005) and 0.44 (95% CI 0.40 to 0.47, p < 0.005), with minimal clinically important difference (MCID) of 10 and 0.18, respectively.^16,19^

**Table II.** Femoral component outcomes.

Outcome	n (%)	Mean months (range)	95% CI	PTIR
Femoral component in situ	512 (99)	23.1 (0.2 to 54.6)	21.6 to 24.6	N/A
Death	3 (0.6)	14.5 (7.1 to 23)	7.1 to 21.9	0.37
Reoperation	5 (1)	5.2 (0.7 to 20)	0 to 11.7	0.61
PJI – DAIR	1 (0.2)	1.5	N/A	0.12
PPF – ORIF	1 (0.2)	20	N/A	0.12
Instability – head exchange	1 (0.2)	2.7	N/A	0.12
Femoral component revision	2 (0.4)	0.9	0.6 to 1.3	0.24
PPF	1 (0.2)	0.7	N/A	0.12
Haematoma	1 (0.2)	1.2	N/A	0.12

DAIR, debridement, antibiotics, and implant retention; N/A, not applicable; ORIF, open reduction internal fixation ; PJI, periprosthetic joint infection; PPF, periprosthetic fracture; PTIR, person-time incidence rate per 100 person-years.

## Discussion

Our results are consistent with previous studies reporting outcomes with the Actis femoral component, which have reported all-cause revision rates of 0.9% at two-year, 0.59 to 1.08% at three-year, and 1.2% at five-year follow-up.^20-23^

Our series represents the largest cohort of Actis femoral components with PROMs to date, and the second to compare pre- and postoperative results. It has previously demonstrated excellent functional outcomes, equivalent to those of the well-established Corail (DePuy Synthes, USA) femoral component, and a significant reduction in POPPF at short-term follow-up when compared against similar uncollared femoral components.^8,22,24^

The design rationale behind the Actis femoral component is intended to provide compatibility across surgical approaches while optimizing stability, reducing the risk of complications such as PPF, and ultimately maximizing survivorship. Its short length (95 to 119 mm), reduced lateral shoulder, and angled insertion features are optimized for use with the anterior approach.^14^ All femoral components in our series were implanted via the posterior approach, similarly to Ricotti et al,^8^ and previous analysis has not shown a difference in clinical or radiological outcome based on surgical approach.^22^

The Actis femoral component is composed of Ti6Al4V titanium alloy with proximal porous coating and plasma-sprayed HA to provide an initial scratch-fit at the bone-implant interface and allow for tissue ingrowth and osseointegration.^14^ Radiological analysis by Hawkins et al^25^ has demonstrated osseointegration in 85% of patients by one-year follow-up.

Triple-tapered geometry limits rotational and axial movement to improve primary stability, while canal preparation using a hybrid-broaching technique increases medial-lateral metaphyseal fill while preserving bone in the anterior-posterior plane to reduce PPF rates.^14,26^

There is a collarless option for the Actis femoral component; however, all femoral components in our series featured a medial collar. This provides significantly greater immediate stability, both vertically and rotationally.^26-28^ Cementless femoral components which incorporate a medial collar in their design have demonstrated advantages over collarless cementless femoral components and polished tapered cemented femoral components in terms of PPF and reoperation rates.^5-11^

Robinson et al^10^ compared the collared Corail femoral component with the Exeter (Stryker Orthopaedics, USA) cemented femoral component and found higher rates of IOPPF with cementless femoral components which did not impact early recovery or PROMs, but no difference in the rate of POPPF, and significantly more reoperation for POPPF with cemented femoral components. Similarly Orce Rodriguez et al^11^ conducted survivorship analysis of the Australian National Joint Registry, and found that all-cause revision was significantly lower for collared cementless femoral components than for cemented femoral components in patients aged 75 years and older.

Our study has several limitations such as its retrospective nature, short interval follow-up, lack of direct comparator, incomplete secondary outcome data, and lack of radiological analysis.

Low event-rates for revision surgery and fracture would necessitate longer follow-up and larger patient numbers for further statistical analysis. Given the retrospective design of this study, randomization was not possible. However, the use of consecutive patients and inclusion of multiple surgeons across multiple centres helps to reduce selection bias.

PROMs were available for 62.5% of patients: this is a major limitation and there are two main contributory elements. First, our series includes 86 femoral components that had not yet reached their six-month review at the time of data collection. Of the 431 femoral components beyond that timepoint, 74.5% had complete reporting of secondary outcome metrics. This deficit may reflect a failure or delay in returning completed questionnaires, a delay in distribution of questionnaires, delays in processing data at a registry level, or a combination of these factors.

Sample size is a relative strength of the current study, with our cohort representing the largest set of Actis femoral components with PROMs in the literature to date.

In conclusion, the Actis femoral femoral component demonstrates excellent functional outcomes which are reproducible across multiple surgeons in non-designer centres using a posterior approach, with low rates of revision surgery and PPF. There are several limitations to this study, however, and further research is warranted to assess implant performance at longer term follow-up. Nevertheless, there is strong evidence to support the use of short collared cementless femoral femoral components, and greater component versatility with tissue-sparing approaches will reduce heterogeneity and improve generalizability in future research.

**Take home message**

- The Actis collared cementless femoral stem demonstrates excellent functional outcomes at short-term follow-up, with low rates of periprosthetic fracture and revision surgery.

## Data Availability

The datasets generated and analyzed in the current study are not publicly available due to data protection regulations. Access to data is limited to the researchers who have obtained permission for data processing. Further inquiries can be made to the corresponding author.
